# Chikungunya virus was isolated in Thailand, 2010

**DOI:** 10.1007/s11262-014-1105-5

**Published:** 2014-08-12

**Authors:** Mikiko Sasayama, Surachet Benjathummarak, Norihito Kawashita, Prasert Rukmanee, Suntaree Sangmukdanun, Promsin Masrinoul, Pannamthip Pitaksajjakul, Orapim Puiprom, Pitak Wuthisen, Takeshi Kurosu, Panjaporn Chaichana, Pannamas Maneekan, Kazuyoshi Ikuta, Pongrama Ramasoota, Tamaki Okabayashi, Pratap Singhasivanon, Natthanej Luplertlop

**Affiliations:** 1 Mahidol-Osaka Center for Infectious Diseases, Ratchathewi, Bangkok, 10400 Thailand; 2 Research Institute for Microbial Diseases, Osaka University, Suita, Osaka 565-0871 Japan; 3 Center of Excellence for Antibody Research, Faculty of Tropical Medicine, Mahidol University, Ratchathewi, Bangkok, 10400 Thailand; 4 Department of Environmental Pharmacometrics, Graduate School of Pharmaceutical Sciences, Osaka University, Suita, Osaka 565-0871 Japan; 5 Department of Tropical Hygiene, Faculty of Tropical Medicine, Mahidol University, Ratchathewi, Bangkok, 10400 Thailand; 6 Department of Virology, Research Institute for Microbial Diseases, Osaka University, Suita, Osaka 565-0871 Japan; 7 Present Address: Department of Microbiology and Immunology, Faculty of Tropical Medicine, Mahidol University, Ratchathewi, Bangkok, 10400 Thailand

**Keywords:** Chikungunya virus, Thailand, Virus replication, Genetic variation

## Abstract

Chikungunya fever (CHIKF) is an acute febrile illness caused by a mosquito-borne alphavirus, chikungunya virus (CHIKV). This disease re-emerged in Kenya in 2004, and spread to the countries in and around the Indian Ocean. The re-emerging epidemics rapidly spread to regions like India and Southeast Asia, and it was subsequently identified in Europe in 2007, probably as a result of importation of chikungunya cases. On the one hand, chikungunya is one of the neglected diseases and has only attracted strong attention during large outbreaks. In 2008–2009, there was a major outbreak of chikungunya fever in Thailand, resulting in the highest number of infections in any country in the region. However, no update of CHIKV circulating in Thailand has been published since 2009. In this study, we examined the viral growth kinetics and sequences of the structural genes derived from CHIKV clinical isolates obtained from the serum specimens of CHIKF-suspected patients in Central Thailand in 2010. We identified the CHIKV harboring two mutations E1-A226V and E2-I211T, indicating that the East, Central, and South African lineage of CHIKV was continuously circulating as an indigenous population in Thailand.

**Electronic supplementary material:**

The online version of this article (doi:10.1007/s11262-014-1105-5) contains supplementary material, which is available to authorized users.

## Introduction

Chikungunya virus (CHIKV; family *Togaviridae*; genus *Alphavirus*) is a mosquito-borne, single-stranded, positive-sense RNA virus that is the causative agent of chikungunya fever (CHIKF), which is a major public health problem in Africa, South America, and Southern and Southeastern Asia [1, 2]. CHIKV was first identified in Tanzania in the 1950s, and recent huge outbreak occurred in Kenya in 2004. This re-emerged epidemic subsequently spread to several countries in the Indian Ocean and India, with documented outbreak in Italy in 2007 [3]. The outbreak in New Caledonia in 2011 and the ongoing outbreak in Caribbean in 2013–2014 raise public health concerns again due to increasing number of CHIKV infections [4, 5]. There are three distinct lineages of CHIKV: the West African genotype; the East, Central, and South African (ECSA) genotype; and the Asian genotype.

In Thailand, outbreaks of chikungunya were first documented in the early 1960s, and the most recent outbreak was reported in 2008–2009 [6]. Classical CHIKF is characterized by high fever, nausea, rash, and severe arthralgia; however, the clinical signs and symptoms of CHIKF are indistinguishable from those of dengue, and both illnesses are transmitted by Aedes mosquitoes, such as *Ae*. *aegypti* and *Ae*. *albopictus* [7, 8].

Studies of the clinical and molecular characteristics of CHIKV during the recent outbreak of chikungunya in Thailand indicated that the virus belonged to the ECSA genotype, which was reported in the Indian Ocean islands after 2005 [9]. These isolates harbored the E1-A226V mutation, which increases virus transmissibility via *Ae*. *albopictus* [10, 11]. By December 2009, CHIKV had spread to the Central, Northeastern, and Northern provinces of Thailand, infecting more than 46,000 individuals [1]. No study of CHIKV clinical isolates in Thailand has been published since 2009; therefore, we need to remain vigilant for the endemic situation of chikungunya in Thailand. Here, we examined the viral growth kinetics and sequences of the structural genes derived from CHIKV clinical isolates obtained from the serum specimens of CHIKF-suspected patients in Ratchaburi Province (100 km west of Bangkok) in 2010.

## Results and discussion

The Royal Thai Government and the Faculty of Tropical Medicine at Mahidol University (MU-TropMed) are currently monitoring emerging diseases (e.g., dengue and malaria) with a view to future control and prevention in Ratchaburi Province. The aim of the present study was to examine the endemic situation regarding CHIKV circulation in Thailand, and in Ratchaburi Province in particular. From August to September, 2010, the MU-TropMed and Health Promoting Hospital (HPH) in Ratchaburi Province obtained 50 serum samples from individuals suspected of harboring CHIKF. Permission regarding the use of anonymous specimens was granted by the Director of HPH, and this study was approved by the Ethics Committee at MU-TropMed (MUTM 2012–047–01). Here, we report the isolation and molecular characterization of seven clinical isolates of CHIKV, which were derived from Thai individuals showing characteristic symptoms.

All serum specimens were tested for CHIKV infection using the SD BIOLINE Chikungunya IgM rapid test kit (Standard Diagnostics, Kyonggi-do, Korea). Briefly, 50 µl of serum sample was dropped into the well of the test device, and assay diluent was subsequently added. Test results were interpreted at 10 min. We also performed real-time reverse transcription-PCR (RT-PCR) assay, targeting the *E1* gene, using the iScript One-Step RT-PCR Kit for Probes (Bio-Rad, CA, USA). Viral RNA was extracted from 140 µl of serum sample using the QIAamp Viral RNA Mini Kit (QIAGEN, Hilden, Germany) according to manufacturer’s instruction. The following primers were used for the real-time RT-PCR: CHIK-forward (AAGCTYCGCGTCCTTTACCAAG), CHIK-reverse (CCAAATTGTCCYGGTCTTCCT), and CHIK-probe (FAM-CCAATGTCYTCMGCCTGGACACCTTT-TAMRA) as described previously [12].

Of the 50 serum samples tested, 6 (12 %) were identified as CHIKV positive by real-time RT-PCR, and one (2 %) was identified as chikungunya positive by both real-time RT-PCR and Chikungunya IgM antibody test kit. All seven CHIKV-positive specimens were obtained from individuals in the acute phase of the illness (≤6 days post-onset). We isolated CHIKV using an *Ae*. *albopictus*-derived cell line, C6/36, and the supernatants of individual clinical isolates were used for further study.

We first examined the viral growth kinetics of two clinical isolates (CP9 and CP11) and three control strains (Ross strain_1953, #32808_2008, and #16856_2009) selected randomly. Vero and C6/36 cells were inoculated with these viruses at a multiplicity of infection of one. The time of inoculation was set as “hour 0”. Culture supernatants were collected at 4, 8, 12, 24, and 28 h post-inoculation, and were titrated with plaque assay. The viral titers of the clinical isolates were significantly higher than those of the controls during the first 8 h post-inoculation in Vero cells (*P* < 0.0005) and the first 12 h post-inoculation in C6/36 cells (*P* < 0.005) (Supplemental Fig. 1). In Vero cells, the peak viral titers for CP9 and CP11 at 12 h post-inoculation were 3.5 × 10^6^ and 3.6 × 10^6^ PFU/mL, respectively. In C6/36 cells, the peak viral titers for CP9 and CP11 were 5.9 × 10^8^ and 4.8 × 10^8^ PFU/mL at 24 h post-inoculation, respectively. We also examined the cytopathic effects (CPEs) of CP9, CP11, and the control viruses against C6/36 cells. Both isolates showed milder CPEs in C6/36 cells than the controls.

**Fig. 1:**
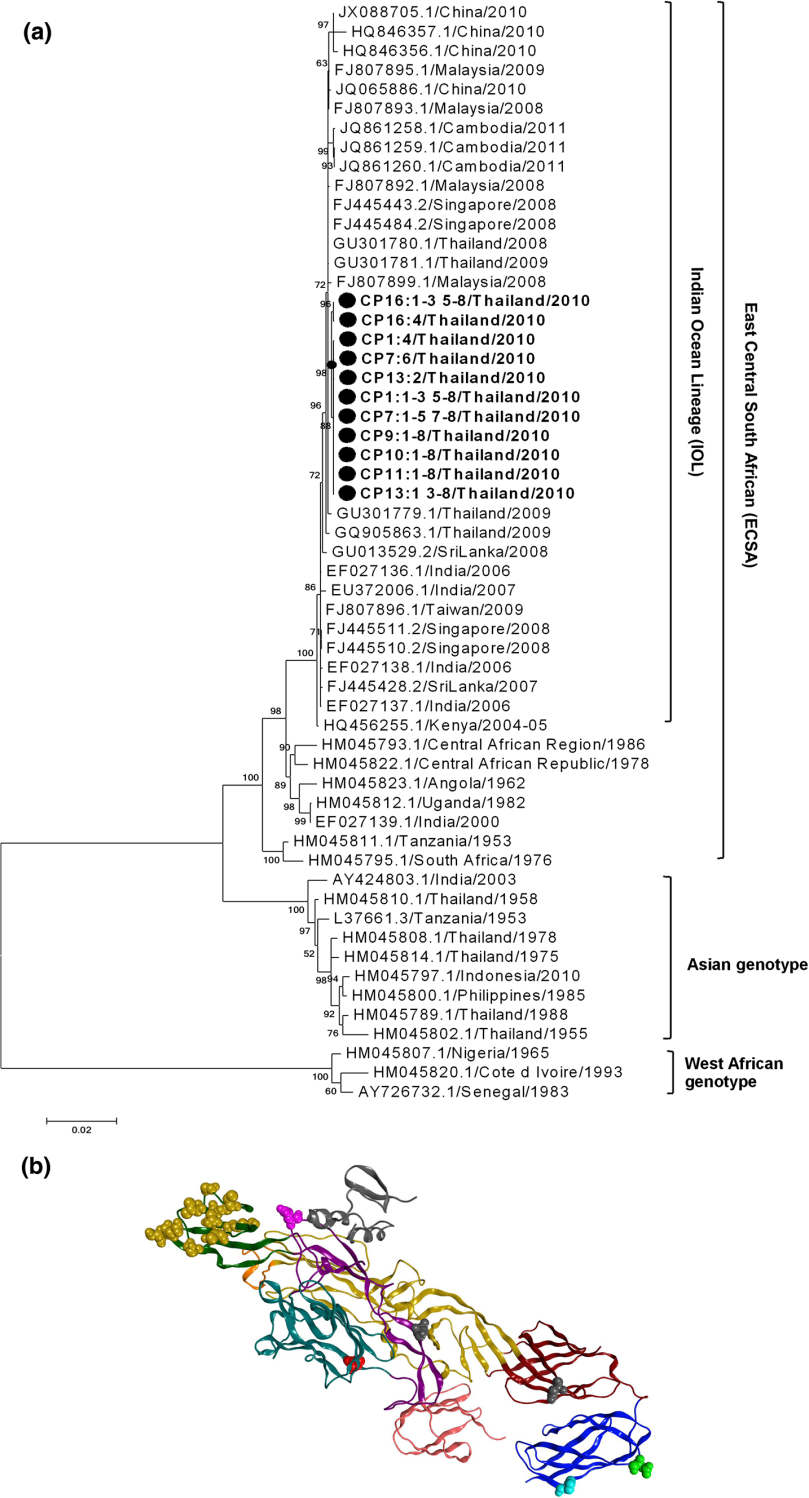
**a** Phylogenetic tree showing the relationships between the structural genes of different chikungunya virus (CHIKV) strains. The information was obtained from 56 clones derived from 7 infected patients in Ratchaburi Province of Thailand in 2010. The numbers below or above the branch nodes represent the neighbor-joining bootstrap values. Analysis was based on the nucleotide sequences of the structural genes using the MEGA 5.05 program and the maximum likelihood method based on 1,000 bootstrap replications. The 2010 isolates are denoted by *black circles* and *bold type*. *Scale bars* indicate 0.02 nucleotide substitutions per site. GenBank Accession numbers for all isolates were recorded. GenBank Accession nos: CP1 (AB857730–AB857737); CP7 (AB857738–AB857745); CP9 (AB857746–AB857753); CP10 (AB857754–AB857761); CP11 (AB857762–AB857769); CP13 (AB857770–AB857777); and CP16 (AB857778–AB857785), **b** Three-dimensional structures of chikungunya E1, E2, and E3 monomers by ribbon model were obtained from the Protein Data Bank (ID code 3N41) [16]. The domains are color coded as follows: Domain I of E1 (*dark red*), domain II of E1 (*dark yellow*), domain III of E1 (*dark blue*), the FL region of E1 (*orange*), domain A of E2 (*blue–green*), domain B of E2 (*dark green*), domain C of E2 (*pink*), the arch region of E2 (*purple*), and E3 (*gray*). Mutated residues are depicted as a space-filling model: H456 (*red*), E572 (*magenta*), P1113 (*light green*), N1158 (*light blue*), and sugar-binding residues (*gray*). Yellow in space-filling model shows the immunodominant site. This figure was prepared by Molecular Operating Environment (Chemical Computing Group Inc) (Color figure online)

Next, to examine sequence variations within the viral genome, the seven clinical isolates were subjected to plaque purification in Vero cells. Eight plaques were selected for each of the isolates: CP1:1–8, CP7:1–8, CP9:1–8, CP10:1–8, CP11:1–8, CP13:1–8, and CP16:1–8. The region containing the structural genes (capsid–E3–E2–6k–E1) was amplified using primers published by Sreekumar et al. [13]. The amplicons were sequenced and aligned with the reference strain sequences available in GenBank (Fig. 1a). The E1-A226V and E2-I211T mutations, which provide a suitable background for CHIKV adaptation in *Ae*. *albopictus* [10, 11, 14, 15], were relatively well conserved among all clones obtained from the seven clinical isolates. Furthermore, several sequence variations were identified. We identified several unique amino acid substitutions that were not present in the strains responsible for the Thai outbreak in 2008–2009: E1-N349I in all CP16 clones; E1-P304L in clone CP7:6; E2-H131Y in clone CP13:2; and E2-E247A in clone CP16:4 (Table 1). These substitutions were conserved when compared to any strains reported in neighboring countries. Moreover, these are exposed on the surface of the molecule, suggesting that it may affect interaction of them with the other E monomers or antibodies (Fig. 1b) [16]. Phylogenetic analysis showed that all of the clinical isolates in 2010 formed a homogeneous cluster within a broad group, which also included the isolates from the Thai outbreak of 2008–2009 (ECSA genotype). Notably, all clones formed a branch that shared >99 % homology with the Indian Ocean Lineage (IOL), a relatively independent cluster within the ECSA genotype that caused an outbreak of unprecedented magnitude in 2005–2006 (Fig. 1a). This conclusion was also supported by the phylogenetic analysis based on *E1* gene sequence (data not shown). The CHIKV isolates from China (2010), India (2010–2011), and Cambodia (2011) also clustered within the IOL.

**Table 1:** Novel amino acid substitutions identified in the 56 chikungunya virus (CHIKV) clones derived from 7 patients

Clinical isolates	Region	Polypeptide position^a^	Protein position^b^	2008-2009 Thai outbreak strain	Amino acid substitution
CP13-2	E2	456	131	H	Y
CP16-4	E2	572	247	E	A
CP7-6	E1	1,113	304	P	L
CP16 (all clones)	E1	1,158	349	N	I

^a, b^Polypeptide and protein positions are in accordance with the published sequence of CHIKV complete genome (GenBank Accession no. AF369024.2)

In conclusion, we isolated CHIKV derived from CHIKF-suspected patients in Ratchaburi Province of Thailand in 2010, and we report that CHIKV shows different characteristics when cultured in C6/36 cells, although it is not clear whether this is owing to the amino acid substitutions in the structural genes. Furthermore, CHIKV quasispecies are present in the Thai isolates from 2010, which include the clones harboring unique amino acid substitutions close to the immunodominant site (Fig. 1b) [16]. However, as we cannot rule out the possibility of antigenic escape mutants, further analysis is required.

If we anticipate new outbreaks of chikungunya in Thailand, then we must increase CHIKV surveillance in disease-epidemic areas and deal with local outbreaks more effectively. Further studies of CHIKV isolated from either mosquitoes or chikungunya patients are needed.

## Electronic supplementary material

Below is the link to the electronic supplementary material.

Supplementary material 1 (TIFF 1491 kb)

Supplementary material 2 (DOCX 11 kb)
