# Supplementary figures and images for: Chikungunya virus was isolated in Thailand, 2010
Source: Virus Genes. 2014 Aug 12;49(3):485–9. doi: 10.1007/s11262-014-1105-5 (PMC4232745; doi:10.1007/s11262-014-1105-5)

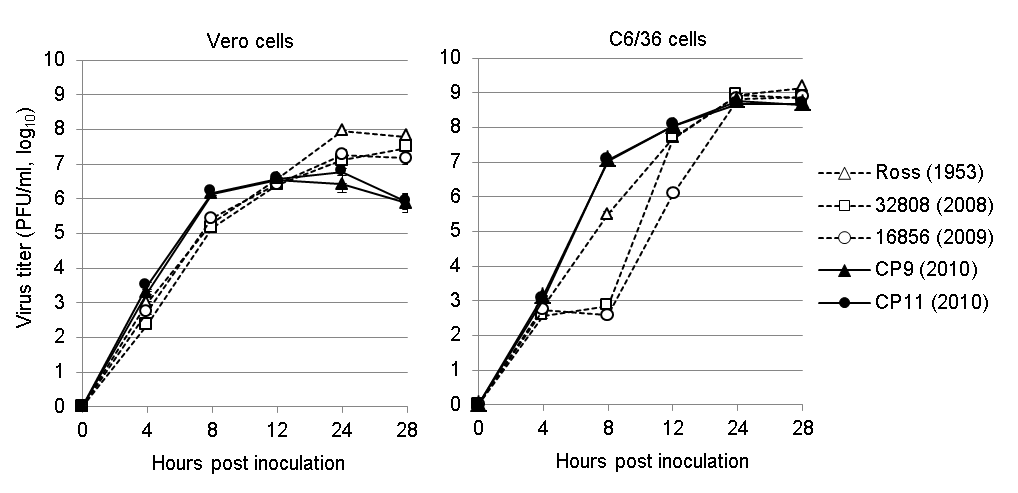

Supplement: Supplementary file 1 — Supplementary material 1 (TIFF 1491 kb) [file 11262_2014_1105_MOESM1_ESM.tif]
