# Supplementary material for: Chikungunya virus was isolated in Thailand, 2010
Source: Virus Genes. 2014 Aug 12;49(3):485–9. doi: 10.1007/s11262-014-1105-5 (PMC4232745; doi:10.1007/s11262-014-1105-5)
Supplement: Supplementary file 2 — Supplementary material 2 (DOCX 11 kb) [file 11262_2014_1105_MOESM2_ESM.docx]

**Supplemental Figure 1 Comparison of chikungunya virus (CHIKV) growth kinetics**

CP9 (▲) and CP11 (●), Ross strain (△), #32808 (□), and #16856 (○) were propagated in Vero cells and C6/36 cells. Cells were inoculated at a multiplicity of infection of 1. The time of inoculation was set as “hour 0”. Culture supernatants were collected at 4, 8, 12, 24, and 28 h post-infection, and the virus titers were assessed in a Vero cell-based plaque assay. CP9 and CP11 were first detected at 4 h post-inoculation in both Vero and C6/36 cultures. The titers of CP9 and CP11 in C6/36 cells were consistently higher than those of the Ross strain, #32808, and #16856 until 12 h post-inoculation in both Vero and C6/36 cells. Overall, the titers of CP9 and CP11 were higher in C6/36 cells than in Vero cells.
